# A Cross-Sectional Analysis of Clinical and Biological Characteristics of Inpatients with Complicated Acute Pyelonephritis

**DOI:** 10.3390/antibiotics15040363

**Published:** 2026-04-01

**Authors:** Marius-Costin Chițu, Carmen-Marina Pălimariu, Teodor Salmen, Tudor-Petru Nicodin, Delia Reurean-Pintilei, Dan-Arsenie Spînu, Viorel Jinga, Anca Pantea Stoian, Dan Liviu Dorel Mischianu

**Affiliations:** 1 Doctoral School of “Carol Davila”, University of Medicine and Pharmacy, 020021 Bucharest, Romania; marius-costin.chitu@drd.umfcd.ro (M.-C.C.);; 2 Infectious Disease Department of Emergency, University Central Military Hospital, 010825 Bucharest, Romania; 3 Cantacuzino National Military Medical Institute for Research and Development, 050096 Bucharest, Romania; 4 Pitesti Emergency County Hospital, 110283 Pitesti, Romania; 5 Urology Department, “Dr. Carol Davila” Central Military Emergency University Hospital, 134 Calea Plevnei, 010825 Bucharest, Romania; 6 Department of Medical-Surgical and Complementary Sciences, Faculty of Medicine and Biological Sciences, Ștefan cel Mare University, 720229 Suceava, Romania; 7 Department of Diabetes, Nutrition and Metabolic Diseases, Consultmed Medical Centre, 700544 Iasi, Romania; 8 Department of Urology, Prof. Dr. Theodor Burghele Clinical Hospital, 061344 Bucharest, Romania; 9 Academy of Romanian Scientists, 050045 Bucharest, Romania; 10 Department of Diabetes, Nutrition and Metabolic Diseases, Carol Davila University of Medicine and Pharmacy, 050474 Bucharest, Romania

**Keywords:** acute obstructive pyelonephritis, antimicrobial-resistant pathogens, antimicrobial resistance, C-reactive protein, D-dimer, sepsis prediction, urinary tract infection, inflammatory biomarkers

## Abstract

**Background**: Complicated acute pyelonephritis (AP) is a severe upper urinary tract infection associated with systemic inflammation, organ dysfunction, and the risk of sepsis. The increasing prevalence of antimicrobial-resistant (AMR) organisms can alter clinical management. This study aimed to characterize the biological profile of inpatients with complicated AP and to eventually identify laboratory markers associated with risks of sepsis and AMR infections. **Material and Methods**: A retrospective observational analysis on 553 adult inpatients diagnosed with complicated AP between 2021 and 2025 was conducted in a tertiary center. Demographic, clinical, and biological parameters were analyzed, including inflammatory markers and renal and hepatic markers. **Results**: Group characteristics included a mean age of 63.82 ± 15.67 years, and 63% were female. At admission, inflammatory markers were raised, with leukocytosis (15.6 ± 5.8 × 10^3^/µL), neutrophilia (10.1 ± 4.7 × 10^3^/µL), and elevated C-reactive protein (CRP) (median 43.2 mg/dL). Coagulation activation was significant with elevated fibrinogen of 747 ± 145 mg/dL and D-dimer with a median level of 1249 ng/mL, of which 58% exceeded 1000 ng/mL. Mild to moderate renal impairment was frequently observed (creatinine 1.69 ± 0.76 mg/dL). In multivariate analysis, no biological parameter proved to be an independent predictor of AMR status among organisms. **Discussion and Conclusions**: Inpatients with complicated AP showed a pronounced inflammatory and procoagulant biological profile that did not vary between AMR pathogen and non-AMR pathogen infections. This suggests that the clinical value of biomarkers, such as leukocyte and neutrophile, CRP, D-dimer, fibrinogen, procalcitonin, urea, and creatinine, lies primarily in assessing disease severity rather than predicting antimicrobial resistance. The microbiological profile was dominated by Gram-negative pathogens, particularly *Escherichia coli*, although a heterogeneous spectrum of microorganisms was identified.

## 1. Introduction

Acute pyelonephritis (AP) is defined as a severe bacterial infection of the upper urinary tract, involving inflammation of the renal parenchyma, most commonly arising ascendingly from the lower urinary tract [1,2]. It can be a potentially organ- and life-threatening condition in cases of timely treatment absence due to the parenchymal destruction, abscess formation, acute kidney injury, and, eventually, progression to systemic sepsis [3,4].

Epidemiologically, AP is a considerable health burden worldwide. In the United States, the annual estimation is 0.5 to 1.1 million episodes, while the global annual estimates is 10 to 26 million cases [5]. A marked adult gender-based disparity has consistently been observed, with females experiencing AP at substantially higher rates than males, with annual incidence rates estimated at 15–17 cases versus 3–4 cases per 10,000 individuals [6]. This discrepancy has been attributed to anatomical and physiological characteristics that can be facilitators for uropathogen ascension, such as shorter urethral length and the proximity of the perineal region to the gastrointestinal tract [7,8].

AP is frequently managed in outpatient settings, but approximately 20% of adult patients require hospitalization for parenteral antimicrobial therapy or management of complications, with increased rates of hospitalization for extreme groups of populations, such as pediatric and elderly populations [1,3]. Furthermore, acute obstructive pyelonephritis (AOP) significantly contributes to severe complications in critically ill individuals. It often causes bloodstream infections or sepsis, originating from a renal focus. These complications account for up to 10% of intensive care unit admissions and result in thousands of deaths annually in the United States [4].

In addition, urinary tract infections (UTIs) are among the most common reasons for antibiotic prescriptions. This significantly contributes to the selective pressure that drives the emergence and spread of antimicrobial resistance [9]. The rise of extended-spectrum β-lactamase (ESBL)-producing strains and antimicrobial-resistant (AMR) non-fermenting pathogens complicates therapy and limits empirical treatment choices [10]. These issues are specifically relevant in complicated UTIs and AP, where the lack of early and effective antimicrobial therapy may lead to severe disease progression [11]. Therefore, tailoring diagnostic and treatment strategies to local resistance patterns is essential, to reduce morbidity, hospital stay, and healthcare-associated costs [12].

The economic burden associated with AP is also significant; direct and indirect costs have been estimated to exceed USD 2 billion annually (in 2000 USD). This reflects the extensive resources required to manage this disease [6,13].

The aim of this study was to analyze the biological characteristics of inpatients with complicated AP, specifically assessing hematological, hepatic, and renal parameters as well as inflammation markers during treatment, to determine whether any of these parameters predict the development of sepsis/septic shock with organ dysfunction. Our key hypothesis is that routine laboratory biomarkers obtained at hospital admission not only reflect disease severity but also differ according to whether infections are caused by AMR pathogens or non-AMR pathogens. We further propose that AMR infections may delay effective therapy, prolong bacterial growth, increase inflammation, and elicit a more pronounced systemic response upon hospital presentation [14,15]. For this study, AMR organisms were defined and classified according to international criteria published by Magiorakos et al. [16], which establish standardized categories based on resistance to multiple antimicrobial agents.

## 2. Results

### 2.1. Group Characteristics

The baseline characteristics of the 553 included patients are summarized in Table 1.

### 2.2. General Inflammatory Profile

A total of 553 adult patients diagnosed with complicated AP were included in the biological analysis.

The pathology underlying complicated AP was obstruction. Its causes were ureteral lithiasis (*n* = 489), malignant obstruction (*n* = 58), and ureteral strictures (*n* = 6), as shown in Table 1.

Upon patient admission, laboratory findings consistently revealed a uniformly intense systemic inflammatory response. This was reflected by marked elevations in leukocyte count, neutrophil predominance, and acute-phase reactants.

The mean white blood cell (WBC) count was 15.6 ± 5.8 × 10^3^/µL. This indicates moderate to severe leukocytosis in most patients. Neutrophils were the dominant leukocyte subtype, with a mean value of 10.1 ± 4.7 × 10^3^/µL. This finding is consistent with an acute bacterial etiology. These results confirm the presence of a robust innate immune response typically seen in upper urinary tract infections (UTIs).

C-reactive protein (CRP) levels were also markedly elevated across the cohort. The median CRP value was 43.2 mg/L. The IQR was 60.54, 94.75 mg/L. This indicates a substantial proportion of patients showed a moderate- to high-grade acute-phase response at hospital admission.

### 2.3. Coagulation and Endothelial Activation Markers

Markers associated with coagulation activation and endothelial dysfunction were also frequently abnormal. Plasma D-dimer levels showed a median value of 1249 ng/mL (IQR: 777–1831 ng/mL); notably, 58% of patients had values exceeding 1000 ng/mL, and approximately 12% exceeded 2000 ng/mL, suggesting a significant activation of the coagulation cascade in a large subset of cases.

Furthermore, fibrinogen levels were consistently elevated, with a mean concentration of 747 ± 145 mg/dL, reinforcing the presence of an acute inflammatory and prothrombotic state. These abnormalities occurred in the absence of documented thromboembolic events, supporting the hypothesis of inflammation-driven coagulation activation rather than overt thrombosis.

### 2.4. Renal and Hepatic Involvement

Examining organ involvement, renal function parameters demonstrated mild to moderate impairment in a relevant proportion of patients. Mean serum urea was 50.4 ± 23.9 mg/dL, while mean creatinine reached 1.69 ± 0.76 mg/dL, suggesting transient renal dysfunction related to infection severity, dehydration, or systemic inflammatory effects.

Consistent with systemic effects, hepatic enzymes exhibited variable but frequently abnormal values. Alanine aminotransferase (ALT) showed a median value of 83 U/L, while aspartate aminotransferase (AST) reached a median of 66 U/L. Gamma-glutamyl transferase (GGT) was also elevated, with a median of 93 U/L. These findings are compatible with reactive hepatic involvement secondary to systemic infection rather than primary hepatocellular disease.

### 2.5. Urinary Sediment and Dipstick Findings

Analysis of urinary findings revealed marked inflammatory changes. Leukocyturia was present in the majority of patients, with a median value of 500 leukocytes/high-power field, indicating active urinary tract inflammation. Nitrite positivity was detected in 71% of cases, supporting the predominance of nitrate-reducing Gram-negative uropathogens.

### 2.6. Biological Differences According to AMR Pathogen Status

Regarding AMR, among the 553 included patients, 109 (19.7%) presented infections caused by AMR pathogens.

A comparison of biological parameters between AMR and non-AMR organisms’ infections is presented in Table 2, providing insight into potential differences in clinical markers by pathogen resistance status.

Overall, inflammatory and coagulation markers showed similar values between the two groups, with no statistically significant differences. Mean CRP values were 119.97 mg/L in AMR pathogens infections compared with 110.37 mg/L in non-AMR pathogen cases (*p* = 0.366). Likewise, D-dimer levels did not differ significantly between groups (1258.48 vs. 1293.81 ng/mL, *p* = 0.610).

Hematological parameters, such as leukocyte and neutrophil counts, also showed comparable values between AMR and non-AMR infections, as did renal function markers, including creatinine and urea levels.

These findings suggest that routine inflammatory and coagulation biomarkers at admission may reflect systemic infection severity but do not clearly differentiate AMR organisms from non-AMR organisms’ infections in this cohort.

### 2.7. Multivariable Analysis

A multivariable logistic regression model was constructed to evaluate whether routinely available biological parameters at admission were associated with the presence of AMR pathogen infections. The model included inflammatory markers (CRP, leukocyte count, neutrophil count), coagulation markers (D-dimer, fibrinogen), renal function parameters (creatinine, urea), and procalcitonin levels, as shown in Table 3.

As shown in Table 3, none of the evaluated biological variables were independently associated with AMR pathogen infections. CRP demonstrated a trend toward lower values in AMR pathogen cases, but this association did not reach statistical significance (OR = 0.9988, *p* = 0.294). Similarly, leukocyte count, neutrophil count, fibrinogen, procalcitonin, D-dimer, creatinine, and urea levels were not significant predictors of AMR pathogen infection in the multivariable model.

These findings suggest that routine laboratory parameters obtained at admission may reflect the systemic inflammatory response but are not reliable predictors of antimicrobial resistance in patients with complicated AP.

### 2.8. Microbiological Spectrum

The microbiological spectrum of pathogens isolated in patients with complicated AP is summarized in Table 4.

Microbiological analysis revealed a heterogeneous spectrum of pathogens responsible for complicated AP in the studied cohort, as shown in Table 4.

*Escherichia coli* (*E. coli*) was the most frequently isolated microorganism, accounting for 46.8% of cases, followed by *Enterococcus faecalis* (13.7%) and *Klebsiella pneumoniae* (11.6%). Other relevant pathogens included *Pseudomonas aeruginosa* (7.2%), *Streptococcus group B* (3.6%), and *Proteus mirabilis* (3.3%).

Less frequently identified microorganisms included *Enterobacter* species, *Morganella morganii*, *Citrobacter* species, and non-fermenting Gram-negative bacilli such as *Acinetobacter baumannii*.

Overall, Gram-negative pathogens accounted for the majority of isolates, consistent with the typical microbiological profile observed in complicated UTIs and obstructive pyelonephritis.

Examples of AMR pathogens include ESBL-producing *E. coli* resistant to third-generation cephalosporins but susceptible to carbapenemes, as well as multidrug-resistant *Klebsiella pneumoniae* strains exhibiting resistance to multiple antibiotic classes.

Empirical antimicrobial therapy was initiated in all patients within the first two hours after presentation and evaluation. The majority of patients received third-generation cephalosporins (80%), followed by β-lactam/β-lactamase inhibitor combinations (~10%), fluoroquinolones (~5%), and carbapenems in a minority of cases. Further, 15 cases (2.71%) required antimicrobial therapy adjusted to microbiological analysis.

## 3. Discussion

The current study evaluated the biological characteristics and the dynamics of hematological, renal and hepatic parameters and inflammatory markers in a group of patients diagnosed with complicated AP, aiming to establish if such markers could serve as predictors of the development of sepsis/septic shock with organ dysfunction and if they may differ between AMR organism and non-AMR organism infections.

Moreover, in addition to the present results regarding complicated AP prognosis, we report, in a paper that is currently under review for publication, data about resistance patterns in AP as part of the same research project.

### 3.1. Systemic Inflammatory Response in Complicated AP

The present study highlights a homogeneous, pronounced inflammatory response among inpatients with complicated AP. Elevated leukocyte counts, neutrophilia, and high levels of acute-phase reactants underscore the systemic nature of this infection, which extends beyond localized renal involvement [17]. Similar inflammatory profiles have been described in hospitalized AOP populations and are associated with increased risk of complications, including sepsis and acute organ dysfunction [18].

Leukocytosis and neutrophilia reflect bone marrow activation by pro-inflammatory cytokines such as interleukin-6 and granulocyte colony-stimulating factor. These mechanisms have been described in severe bacterial infections and sepsis-associated syndromes. Consistent neutrophil predominance in this cohort matches prior studies, which emphasizes the diagnostic and prognostic value of neutrophil-based indices in severe UTIs [19]. The neutrophil-to-lymphocyte ratio serves as an easy, available, early marker of sepsis. Although not explicitly calculated in the present study due to incomplete patient files, this ratio can be inferred to be elevated, given the degree of neutrophilia and systemic inflammation [19]. Reports identify neutrophil-to-lymphocyte ratio values above 5 as suggestive of sepsis or impending septic shock, especially in obstructive and complicated AP, supporting its value as an early risk stratification tool [20,21].

Although the neutrophil-to-lymphocyte ratio has been proposed as an early marker of sepsis in AP, this parameter was not systematically calculated in our retrospective dataset because lymphocyte counts were not consistently available for all patients. Therefore, neutrophil-to-lymphocyte ratio analysis could not be reliably performed in the present cohort.

### 3.2. CRP and Fibrinogen as Markers of Disease Severity

CRP elevation was observed in most patients. Levels frequently surpassed thresholds for uncomplicated UTIs. CRP production is driven by interleukin-6 and indicates infection burden and tissue injury [22]. In this cohort, CRP values above 60–90 mg/L were common, indicating moderate to severe inflammation [17,23]. Resistant pathogens are often associated with the delayed initiation of effective therapy, prolonged bacterial replication, and heightened host inflammatory responses, all of which may elevate CRP concentrations [17].

Fibrinogen elevation further supports this observation. Fibrinogen is both an acute-phase reactant and a key coagulation factor. It serves as a molecular link between inflammation and thrombosis. The consistently elevated fibrinogen levels in this study suggest inflammatory hypercoagulability. This phenomenon is recognized in severe infections [24,25,26].

Serum procalcitonin levels are significantly elevated in bacterial AP. These levels correlate with infection severity and systemic complications, including sepsis. In complicated AP, procalcitonin has independent prognostic value. Higher levels are linked with unfavorable clinical outcomes and the need for urgent urological interventions. As in infants and children, procalcitonin is a useful biomarker for distinguishing AP from lower UTIs. It also has superior sensitivity to traditional inflammatory markers in detecting renal parenchymal involvement [27,28,29].

Building on this, multiple studies have demonstrated that elevated procalcitonin levels are independently associated with septic shock, need for urgent urological drainage, and are linked with unfavorable clinical outcomes [27].

In AOP, procalcitonin outperforms traditional markers in predicting progression to sepsis under Sepsis-3 criteria [30]. Moreover, its utility extends across age groups, from pediatric to adult populations, in differentiating upper from lower UTI. Accordingly, it was reasonable to hypothesize that the levels of procalcitonin would be higher in infections caused by AMR pathogens, as these elevated levels might reflect delayed treatment and prolonged bacterial activity [26].

In our cohort, procalcitonin levels were elevated in many patients. However, they did not significantly differ between AMR and non-AMR infections. This finding indicates that procalcitonin reflects the presence and severity of infection, rather than the organism AMR profile. Therefore, procalcitonin may help identify patients with severe systemic infection [27]. Its role in predicting antimicrobial resistance appears limited. Emerging biomarkers, such as presepsin and urine neutrophil gelatinase-associated lipocalin (uNGAL), have also gained attention. uNGAL, in particular, is proposed as a marker of renal parenchymal involvement and tubular injury in AP. It correlates with disease severity and renal dysfunction [31]. These markers may complement traditional laboratory parameters in multimodal risk stratification models.

### 3.3. D-Dimer Elevation and Prothrombotic Implications

In AP, bacterial endotoxins and inflammatory mediators promote tissue factor expression and fibrin formation, resulting in increased fibrinolysis and D-dimer release. The high prevalence of D-dimer elevation is among the most notable biological findings. Although D-dimer has traditionally been associated with venous thromboembolism, its elevation in acute infections is increasingly interpreted as indicative of systemic coagulation activation and endothelial dysfunction. These observations suggest that AP induces inflammation-driven coagulation activation rather than overt thrombosis.

Over half of the patients exhibited D-dimer values above 1000 ng/dL. This finding emphasizes the need for clinicians to recognize complicated AP as a systemic inflammatory condition with prothrombotic risk, even in the absence of overt thrombosis [18]. Similar in sepsis and severe infections, D-dimer indicates endothelial dysfunction and disease severity, underscoring its role in clinical assessment rather than as a specific marker for thrombosis [32].

The clinical relevance of these findings lies in recognizing AP as a condition with potential prothrombotic implications, particularly in elderly patients or those with comorbidities. While routine anticoagulation cannot be recommended based solely on D-dimer elevation, heightened vigilance for thromboembolic complications may be warranted in high-risk subsets [23].

### 3.4. Renal and Hepatic Dysfunction as Secondary Targets

Previous studies indicate that renal function parameters, such as creatinine and urea, are frequently elevated in cases of severe infection or delayed drainage of urinary obstruction [33]. In our cohort, elevated renal function markers were commonly observed, yet no statistically significant differences emerged between AMR and non-AMR infections. This finding could be due to renal impairment in AOP being more strongly associated with the extent and duration of obstruction rather than the AMR profiles of causative pathogens [34,35]. Accordingly, in the acute setting, renal function parameters may more accurately reflect the obstructive aspect of the disease than its microbiological characteristics [34].

Hepatic enzyme elevations followed a reactive pattern, indicating cytokine-mediated hepatocellular stress rather than direct hepatic infection. Recognizing this pattern is clinically relevant, as similar enzyme elevation has been observed in systemic bacterial infections and sepsis-related inflammation [36,37], helping guide differential diagnosis and management decisions.

### 3.5. Biological Predictors of AMR Organism Infections

Patients with AMR pathogens had higher inflammatory markers, supporting the hypothesis that resistant infections cause more severe systemic responses [33]. Additionally, multivariable analysis showed that male gender was linked to AMR organism status, consistent with previous reports connecting male anatomy, prostatic involvement, and delayed healthcare presentation to higher resistance risk [38].

A key objective of this study was to assess whether common biomarkers could identify patients with infections from AMR organisms. Our findings showed no significant differences in inflammatory or coagulation markers between AMR and non-AMR infections. Further, multivariate logistic regression analysis did not identify any biological parameter as an independent predictor of AMR status. Although resistant infections are generally linked to delayed treatment and potentially worse outcomes, their admission biomarkers do not appear to differ substantially from those of susceptible infections.

The more intense inflammatory and coagulation profiles seen in AMR pathogen infections highlight the need for early recognition and suitable empirical therapy. The 2024 IDSA guidance on antimicrobial-resistant Gram-negative infections highlights individualized risk assessment and early escalation in patients with severe presentations or resistance risk factors [39].

### 3.6. Microbiological Profile of Complicated AP

The microbiological distribution in our cohort matches the known epidemiology of complicated UTIs: *E. coli* was the dominant pathogen, responsible for nearly half of all cases [40], consistent with previous studies reporting *E. coli* as the main cause in both uncomplicated and complicated UTIs [41]. Still, a notable proportion of infections were due to other microorganisms, especially *Enterococcus* species, *Klebsiella pneumoniae*, and *Pseudomonas aeruginosa*, reflecting the polymicrobial and varied nature of obstructive UTIs [42].

The presence of non-fermenting Gram-negative bacilli and other opportunistic pathogens underscores the complexity of hospitalized patient populations and the growing burden of healthcare-associated infections. This diversity highlights the need for early microbiological testing and tailored antimicrobial therapy in patients with complicated AP [43].

### 3.7. Clinical Implications

Elevated values at admission may prompt clinicians to anticipate complicated disease courses, prolonged hospitalization, or the need for broader empirical antimicrobial coverage [40].

Although the presence of a systemic inflammatory response in AP is well established, the novelty of the present study lies in the integrated analysis of inflammatory, coagulation, renal and microbiological parameters in a large cohort of patients with complicated AP, with a specific focus on AMR.

Importantly, our findings show that routinely available biological markers at hospital admission do not reliably distinguish AMR from non-AMR infections. This challenges the assumption that resistant infection is necessarily present with a more pronounced inflammatory profile, as suggested by studies focusing on outcomes rather than biological markers [44,45].

These results provide clinically relevant insights. Early identification of AMR cannot rely solely on standard laboratory parameters. It must also incorporate microbiological testing and clinical risk factors.

An important finding of our study is that all patients received empirical antimicrobial therapy promptly after hospital presentation. This early treatment may explain why inflammatory biomarkers did not differ significantly between AMR and non-AMR infections, as prompt therapy could have attenuated the systemic inflammatory response. This observation highlights the importance of early antibiotic administration in complicated AP and suggests that, where treatment is rapid, biological markers may not differentiate resistant from non-resistant infections.

### 3.8. Limitations

This study has several limitations. We did not evaluate the microbiological assessment of patients. Its retrospective single-center design may limit generalizability and the availability of detailed clinical data. This includes the duration of symptoms prior to presentation and the precise timing of effective antimicrobial therapy. We identified a relatively small number of AMR organism infections, despite having a relatively large cohort. Clinical outcome data, such as septic shock, intensive care unit admissions, or mortality rates, were not systematically evaluated. This may have reduced statistical power for detecting subtle differences between groups. This study focused only on patients’ initial status, specifically their first 72 h of treatment and not on their final outcome.

A limitation is the lack of collected data regarding the neutrophil-to-lymphocyte ratio, uNGAL, and D-dimer. We will consider this for our future projects. Nevertheless, the large dataset and comprehensive biological profiling provide valuable insights into the systemic inflammatory characteristics of complicated AP and their potential clinical implications. Future research should integrate classical inflammatory markers and emerging biomarkers into predictive models or nomograms, as recently proposed for complicated AP-related sepsis [38,41]. Prospective multicenter studies should validate such models and define standardized cut-off values for routine clinical practice.

## 4. Materials and Methods

### 4.1. Study Design

A retrospective cross-sectional observational analysis was conducted according to the Helsinki Declaration Recommendations. Also, the study received the Institutional Ethics Committee of the “Dr. Carol Davila” Central Military Emergency University Hospital, Registration number 853/11.02.2026.

### 4.2. Study Setting

Inpatients diagnosed with complicated AP and consecutively admitted between 1 January 2021 and 31 December 2025 to the Inpatient Urology and Infectious Disease Department of Emergency University Central Military Hospital were enrolled. They were monitored only for their initial status and the first 72 h of treatment, not for their final outcome. No deaths occurred in this group during this time.

### 4.3. Study Participants

The inclusion criteria included adults of all ages, both genders, with both community-acquired and hospital-acquired complicated AP. Complicated cases included patients with acute obstructive pyelonephritis, structural urinary tract abnormalities, or other clinical conditions requiring hospitalization.

The exclusion criteria included the pediatric population, patients with incomplete data, patients without complicated AP including AOP.

AMR pathogens were defined as microorganisms harboring resistance mechanisms identified through microbiological testing, including resistant genes or phenotypic resistance patterns. Examples include ESBL-producing *E. coli*, carbapenem-resistant *K. pneumoniae* and multidrug-resistant *P. aeruginosa.*

Patients’ data were collected retrospectively from the electronic registry and included demographic (age, gender), clinical (temperature, blood pressure (BP), heart rate (HR)) and paraclinical WBC count, neutrophil, fibrinogen, D-dimers levels, CRP, procalcitonin, creatinine, urea, transaminase, electrolytes, leukocyturia and nitrites) and data on empirical antimicrobial therapy, including the timing of administration and the antibiotic class administered.

**Figure 1:**
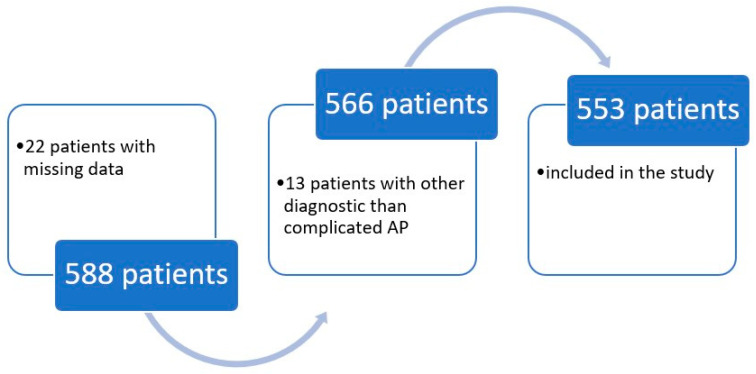
Flowchart of the included patient’s selection. AP—acute pyelonephritis.

After evaluating the 588 included patients against the inclusion and exclusion criteria, 22 had missing data and 13 had a diagnosis other than complicated AP. The data from the remaining 553 patients were evaluated, as shown in Figure 1.

### 4.4. Quantitative Variables

The dataset was cleaned and restructured to identify relevant variables, including gender, age, temperature, BP, HR, WBC count, neutrophil, fibrinogen, D-dimers levels, CRP, procalcitonin, creatinine, urea, transaminase, electrolytes, leucocyturia and nitrites.

### 4.5. Statistical Analysis

Data were collected and structured in Excel databases and analyzed using both Excel and Gnu PSPP 1.4.1 software.

The results were reported as means (standard deviations) or as median (interquartile range). The distribution of data was tested using the Kolmogorov–Smirnov test. In correlation analysis, univariate Pearson or Spearman coefficients were selected depending on variables’ distribution and multivariate regression for identifying independent predictors. Statistical significance tests used Student’s method (*t*-test) or Mann–Whitney U tests, while ANOVA was used to compare means. All statistical tests were two-tailed, and a *p*-value less than 0.05 was considered statistically significant.

## 5. Conclusions

In this cohort of hospitalized patients with complicated AP, elevated inflammatory and coagulation markers were common at admission, reflecting a pronounced systemic inflammatory response; however, these routine biomarkers did not differ significantly between infections caused by AMR organisms and those caused by non-AMR organisms. Their clinical value may, therefore, lie primarily in assessing disease severity rather than predicting antimicrobial resistance.

Gram-negative pathogens, particularly *E. coli*, dominated the microbiological profile. A heterogeneous spectrum of microorganisms was also identified. AMR pathogens were present in a subset of patients, but these were not associated with significantly different levels of routine inflammatory biomarkers at admission. Future prospective studies that integrate clinical parameters, microbiological data, and advanced biomarkers may further improve early risk stratification in patients with complicated AP.

## Figures and Tables

**Table 1:** The baseline characteristics of the included patients.

Characteristic	*n* = 553
Age (mean ± SD)	63.82 ± 15.67 years
Female gender	63%
Obstruction causes	
- ureteral lithiasis, *n*, %	489, 88.42%
- malignant obstruction, *n*, %	58, 10.49%
- ureteral strictures, *n*, %	6, 1.09%
WBC (mean ± SD)	15,608.64 ± 5815.80/dL
Neutrophile (mean ± SD)	10,082.82 ± 4717.65/dL
CRP (median, (IQR))	43.2, (60.54, 94.75) mg/L
D-Dimer (mean ± SD)	1286.84 ± 638.19
Fibrinogen (mean ± SD)	747.06 ± 145.28
Creatinine (mean ± SD)	1.69 ± 0.76 mg/dL
Urea (mean ± SD)	50.36 ± 23.90 mg/dL
TGO/AST (mean ± SD)	68.08 ± 34.58 mg/dL
TGP/ALT (mean ± SD)	80.13 ± 40.43 mg/dL

SD—standard deviation; IQR—interquartile range; WBCs—white blood cells; CRP—C-reactive protein; TGO/AST—aspartate aminotransferase; TGP/ALT—alanine aminotransferase.

**Table 2:** Biological parameters according to AMR organisms’ status.

Parameter	AMR Pathogens (*n* = 109)	Non-AMR Pathogens (*n* = 444)	*p* Value
Leukocytes (cells/µL)	15,789.61 ± 5515.49	15,564.22 ± 5892.33	0.707
Neutrophils (cells/µL)	10,314.15 ± 4441.86	10,026.02 ± 4786.02	0.551
CRP (mg/L)	110.37 ± 95.62	119.97 ± 111.51	0.366
Procalcitonin (ng/mL)	5.48 ± 2.46	5.68 ± 2.67	0.472
D-dimer (ng/mL)	1258.48 ± 649.94	1293.81 ± 635.83	0.610
Fibrinogen (mg/dL)	747.36 ± 155.46	746.99 ± 142.87	0.982
Creatinine (mg/dL)	1.63 ± 0.74	1.71 ± 0.77	0.300
Urea (mg/dL)	51.39 ± 23.99	50.11 ± 23.90	0.618

AMR = antimicrobial-resistant, CRP = C-reactive protein.

**Table 3:** Multivariable logistic regression analysis for predictors of AMR pathogen infection.

Parameter	Odds Ratio	95% Confidence Interval	*p* Value
Leukocytes	0.99999	0.99994–1.00005	0.845
Neutrophils	1.00002	0.99995–1.00009	0.587
C-reactive protein	0.9988	0.9965–1.0011	0.294
Procalcitonin	0.970	0.895–1.052	0.460
D-dimer	0.99988	0.99954–1.00022	0.480
Fibrinogen	1.00008	0.99862–1.00153	0.917
Creatinine	0.861	0.642–1.154	0.317
Urea	1.0047	0.995–1.014	0.339

**Table 4:** Microbiological spectrum of pathogens isolated in patients with complicated AP.

Pathogen	*n*	%
*Escherichia coli*	259	46.8
*Enterococcus faecalis*	76	13.7
*Klebsiella pneumoniae*	64	11.6
*Pseudomonas aeruginosa*	40	7.2
*Streptococcus group B*	20	3.6
*Proteus mirabilis*	18	3.3
*Enterococcus faecium*	13	2.4
*Enterobacter cloacae*	12	2.2
*Morganella morganii*	9	1.6
*Klebsiella oxytoca*	5	0.9
*Citrobacter koseri*	4	0.7
*Staphylococcus aureus*	4	0.7
*Klebsiella aerogenes*	3	0.5
*Candida tropicalis*	3	0.5
*Acinetobacter baumannii*	3	0.5
*Citrobacter freundii*	3	0.5
*Other pathogens*	17	3.1

## Data Availability

Data supporting reported results can be obtained on request from the authors, due to the fact that this is an ongoing doctoral project that still has results to be published.

## Abbreviations

The following abbreviations are used in this manuscript:

ALT	Alanine aminotransferase
AMR	Antimicrobial-resistant
AOP	Acute obstructive pyelonephritis
AP	Acute pyelonephritis
AST	Aspartate aminotransferase
BP	Blood pressure
CRP	C-reactive protein
*E. coli*	*Escherichia coli*
ESBL	Extended-spectrum β-lactamase
GGT	Gamma-glutamyl transferase
HR	Heart rate
uNGAL	Urine neutrophil gelatinase-associated lipocalin
UTIs	Urinary tract infections
WBC	White blood cells
